# High‐Speed Raman Readout of Single Polypeptides via Plasmonic Nanopores

**DOI:** 10.1002/adma.202504436

**Published:** 2025-07-09

**Authors:** Foroogh Khozeymeh Sarbishe, Kirill Khabarov, Maria Blanco Formoso, Ilaria Micol Baldi, Veronica Storari, Henri Haka, Massimo Mastrangeli, Francesco Difato, Andrea Armirotti, Federica Villa, Francesco Tantussi, Francesco De Angelis

**Affiliations:** ^1^ Italian Institute of Technology Genoa 16162 Italy; ^2^ Department of Physics University of Genoa Genoa 16146 Italy; ^3^ Dipartimento di Elettronica Informazione e Bioingegneria Politecnico di Milano Milano 20133 Italy; ^4^ Department of Microelectronics Delft University of Technology Delft 2628CD The Netherlands

**Keywords:** nanopore, plasmonic, polypeptide, Raman, single molecule

## Abstract

The ability to identify individual protein molecules using Surface‐Enhanced Raman Scattering (SERS) spectroscopy, without the need for labelling, is a significant advancement in biomedical diagnostics. However, the inherently small Raman scattering cross‐section of most (bio) molecules necessitates significant signal amplification for successful detection, particularly at the single‐molecule level. A novel approach is introduced for fabricating plasmonic nanopores suitable for sequential Raman readout, namely the recording of Raman spectra from portions of molecules, which are progressively flowing into plasmonic hot spots. The method is based on Capillary‐Assisted Particle Assembly (CAPA)of gold nanoparticles (Au NPs), thus ensuring high stability and cost‐effectiveness. By electrophoretically driving polypeptides through these nanopores, real‐time Raman detection is achieved with a Single‐Photon Avalanche Diode (SPAD) camera, attaining single‐molecule detection at 1 nm concentration with 100 microsecond resolution. Statistical analysis of translocation times, photon scattering rates, and spectral data confirms a linear correlation between dwell time, molecular length, and Raman signal intensity. On average, a photon scattering of 6 photons/amino acid and a translocation time of 7 µs per amino acid is recorded. These results demonstrate the feasibility of sequential Raman readout, overcoming key limitations of SERS. This method represents a significant step toward label‐free, high‐resolution molecular identification, with future potential applications in protein identification.

## Graphical abstract

A label‐free method is presented for single‐protein investigation using plasmonic nanopores that enable stable, cost‐effective Raman detection of polypeptides in real time. An average of 6 photons is measured during a dwell time of 7 microseconds per amino acid. The linear correlations are observed between dwell time, molecular length, and signal, suggesting the feasibility of sequential Raman readout.

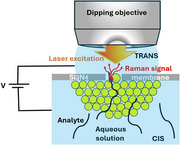

## 1 Introduction

Surface Enhanced Raman Scattering (SERS) and related technologies have attracted the interest of a huge community for nearly three decades.^[^ ^1^, ^2^ ^]^ Such wide and long‐standing interest arises from SERS's ability to achieve sub‐molecular detection sensitivity,^[^ ^3^ ^]^ allowing outstanding discriminating power. In principle, the so‐called Raman fingerprint can distinguish practically any functional group or even different vibrational modes of the same chemical bond. In this regard, SERS has no comparison with other analytical techniques. However, major obstacles prevented the practical applications of SERS, including: i) the difficulty in producing efficient nanostructured substrates with high reproducibility and low costs; ii) the need to adsorb or bind the analyte to plasmonic surfaces; iii) the complex spectral analysis required to identify molecules, hindering the actual possibility of fully exploiting the Raman fingerprint. For example, when analyzing a Raman spectrum, it is relatively straightforward to associate a specific Raman peak with a specific chemical bond or functional group. However, peak association only indicates the presence of a specific functional group within the molecular structure without determining where such a moiety is located. In other words, Raman may inform about molecular composition but not, or only very loosely, about molecular structures; therefore, it is very difficult to identify the molecule in practical cases. This is particularly relevant for polymeric biomolecules such as proteins, DNA, and RNA in which the constituents are largely the same and only their relative spatial coordination makes the difference, together with the total length of the molecule.

The ideal solution would involve recording Raman spectra sequentially, following the molecular order of the moieties along the polymeric chain, which would enable unambiguous molecular identification. This sequential readout concept has been successfully developed in the domain of DNA sequencing using biological nanopores and electrical readouts.^[^ ^4^ ^]^ In those approaches, DNA molecules pass through a nanopore, and the electrical current is measured to identify the specific nucleotide in the pore at any given moment. However, the electrical readout lacks the sophisticated discriminating capability of the Raman fingerprint.^[^ ^5^ ^]^ If a similar sequential approach could be integrated with SERS and plasmonic pores, it could revolutionize the field, especially given the superior ability of Raman fingerprinting to identify specific functional groups. Nevertheless, significant obstacles persist. First, the difficulty of producing plasmonic pores suspended on passing through membranes with a size approaching the molecular scale, namely few nm. Second, SERS is known to be affected by spectral fluctuations in both intensity and position. Third, molecular translocation through nanopores occurs on microsecond timescales, a realm beyond the current capabilities of SERS^[^ ^6^ ^]^ even when using strong emitters such as fluorophores. Small units like amino acids with ordinary Raman cross‐sections produce weak signals, further plagued by fluctuations that undermine the Raman fingerprint advantage. All these challenges make the idea of Raman sequencing apparently distant. Nevertheless, in a set of recent papers, it has been demonstrated that SERS identification of single amino acids and nucleotides is possible at sub‐single molecule levels, namely portions of the single molecules corresponding to single amino acids and nucleotides can be identified.^[^ ^7^, ^8^, ^9^ ^]^ Even single‐point mutations have been successfully investigated.^[^ ^10^, ^11^ ^]^

In this work, we make an advancement toward the development of Raman sequential readout strategies by making two important steps. First, we introduce an easy and affordable fabrication approach for nanopores. We exploit Capillary‐Assisted Particle Assembly (CAPA) of gold nanoparticles (Au NPs) combined with lithographic techniques, to develop a novel nano‐pore fabrication protocol both scalable and affordable. Second, by using electrophoresis, we carried out translocation experiments, and we measured both the translocation time and Raman scattering rate for three different polypeptides. To reach short temporal resolution, we integrated a Single‐Photon Avalanche Diode (SPAD) camera within a commercial Raman spectrometer. This was particularly helpful both for achieving time resolution on the microsecond scale and for counting the number of photons scattered. The former is necessary to monitor the short translocation of molecules in the nanopores, while the latter is essential to quantitatively estimate the number of Raman photons at the single amino acid level. We measure an average translocation time of 7 microseconds per amino acid, very similar for all the investigated amino acids. This value is 15 times longer than that previously reported for solid‐state nanopores.^[^ ^12^ ^]^ In contrast, the number of detected photons is very different for the investigated amino acids because of their distinct Raman cross‐sections. However, thanks to the achieved single‐photon sensitivity, we could robustly detect and estimate an average of 6 photons per amino acid within the translocation time scale. It should be mentioned that the current upper‐bound optical efficiency of our setup is estimated to be 7%. This is primarily due to the quantum efficiency of the SPAD camera that in our case is 20% at λ = 633 nm, the transmission efficiency of ≈90% for the optical system, and the fact that only ≈40% of the Raman scattered photons fall within the currently collected Stokes wavenumber range relevant for organic molecules. Hence, the real number of photons scattered per amino acid is in the order of 85 per translocation event. Such a large number supports that the strategy of sequential readout can enable Raman identification of proteins. Before moving to the technical discussion, we note that the current study introduces significant advances with respect to those pioneering works in the field of extreme sensing by SERS that partially resemble the concept of sequential readout. Examples in this regard are: i) the work on “picocavities” by the groups of Baumberg and Aizpurua^[^ ^13^, ^14^ ^]^ that showed extreme spatial resolution; ii) works on ultrafast SERS dynamics by the group of Brolo^[^ ^15^ ^]^; and iii) SERS sequencing attempts with nanopipettes by the group of Wang,^[^ ^10^ ^]^ and plasmon‐enhanced photochemical reactions at sub‐molecular level^[^ ^16^ ^]^ by the group of Schull.

## 2 Results and Discussion

### 2.1 The Concept

**Figure 1:**
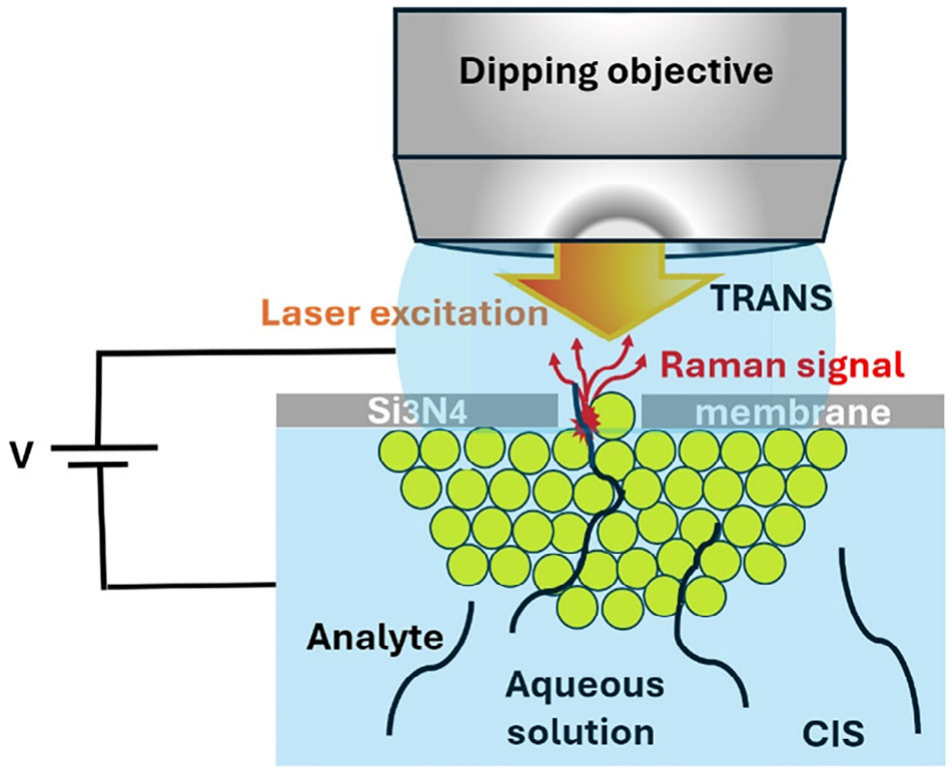
Schematic illustration of the flow‐through electro‐plasmonic setup allowing analyte molecules to be transported across the nanometric pores between the Au NPs and micrometric hole walls. Laser excitation at 633 nm is also indicated, showing the hotspot and collected Raman signal for analyte detection.

The concept is illustrated in **Figure** **1**. As described in more detail in the following section, micrometric pores are fabricated on commercial silicon nitride (Si_3_N_4_) membranes. Capillary forces are then used to deposit a controlled amount of Au NPs onto the membrane, effectively plugging the micrometric pores and creating an intricate network of nanometric pores. A laser beam excites plasmonic hot spots at the interface between the top layer of Au NPs and the electrolyte solution. Analyte molecules are transported through the nanopores from the **CIS** (bottom) to the **TRANS** (upper) chamber via electrophoresis. During translocation, molecules navigate the intricate pore pathway and, upon reaching the final gold layer, are optically excited by the plasmonic hot spots.

This continuous molecular flow through the pores and subsequent interaction with the plasmonic hot spots generate the sequential scattering of Raman photons. As anticipated, a key challenge in fully developing the sequential readout strategy is understanding the translocation time of molecules in relation to the Raman scattering rate and the temporal resolution of Raman spectrometers. The molecule must scatter a sufficient number of photons above the noise level during its translocation through the hot spot. Simultaneously, the Raman spectrometer must have adequate temporal resolution to capture such a short and weak signal. These aspects, along with the fabrication and characterization of plasmonic pores, will be the primary focus of this manuscript.

### 2.2 The Fabrication and Characterization of Nanopores

The fabrication process involves two key steps: i) creating nanohole arrays with a 190 nm diameter on a Si_3_N_4_ membrane and ii) depositing Au NPs into the holes to plug them, forming plasmonic gaps only a few nanometres wide. The latter offers ultra‐narrow pathways containing multiple plasmonic hot spots for the molecule traversing the nanopores. To avoid confusion, we call the 190 nm apertures “holes,” while the plasmonic gaps will be indicated as nanopores or simply gaps. Given that the hole size exceeds 100 nm, several cost‐effective techniques, such as nanosphere lithography or nanoimprinting, can be employed. However, for simplicity, we fabricated the hole array using focused ion beam (FIB) milling.

We preliminarily deposited a 10 nm‐thick titanium adhesion layer, followed by a 20 nm‐thick gold layer onto a 100 nm‐thick Si_3_N_4_ membrane. This is necessary for FIB processing, but it is also useful to screen light and selectively excite plasmonic nanogaps within the holes. Then we milled 5 × 5 arrays of nanoholes in square and hexagonal patterns. After different trials, we chose an optimal diameter of 190 nm. However, the particle deposition is not particularly sensitive to this parameter, thus making the process robust.

**Figure 2:**
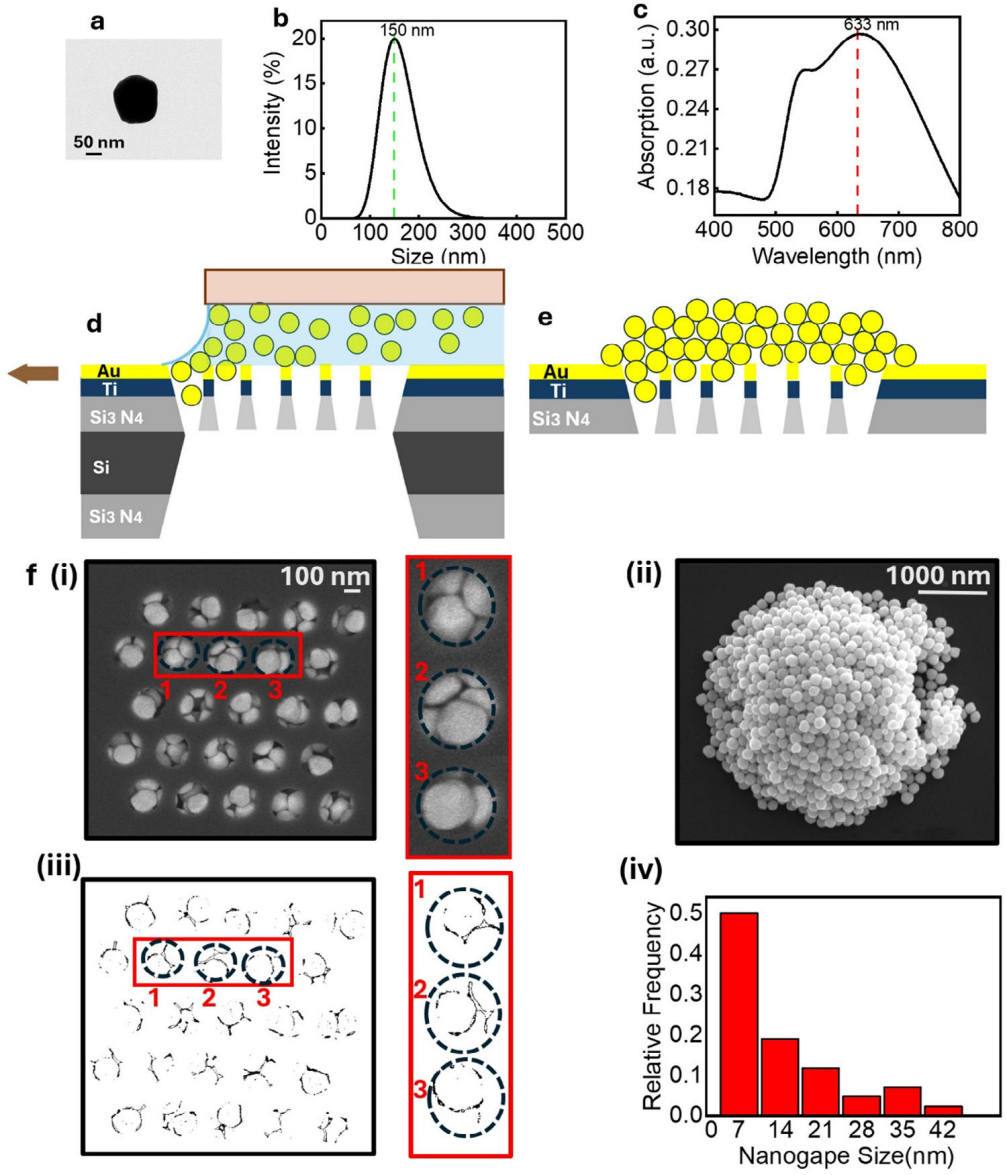
Characterization and fabrication of the SERS substrate. a) TEM image of the Au NPs, b) DLS measurement showing the size distribution of 150 nm Au NPs dispersed in water, c) UV–Vis spectra of 150 nm Au NPs in aqueous solution with high absorption ≈633 nm, d) cross‐sectional schematic of CAPA mechanisms based on particle confinement at the receding contact line of a droplet. As the substrate moves relative to the fixed glass slide, the liquid meniscus at the interface generates capillary forces that drive the Au NPs into the nanoholes, e) Schematics of the central membrane including the nanohole array after running the CAPA experiment. f (i) Front‐side SEM image of a nanohole array‐based device, showing self‐assembled Au NPs strategically positioned within the nanohole array region at the center of the membrane. Insets 1, 2, and 3 display the nanogaps, which serve as passageways for polypeptide analytes through the AuNPs trapped in the nanohole zone. f (ii) SEM image of the device from the back side. f (iii) the FFT‐transformed representation of the SEM image in f (i), showing the transverse nanogaps formed, and f (iv) Histogram of nanogap sizes, confirming the sub‐10 nm pore size.

We employed CAPA to position multiple particles within the nanohole array region. To perform this, a commercial Capillarity‐Assisted particle deposition system from Nanoversa,^[^ ^17^ ^]^ was employed. A cross‐sectional schematic of the particle deposition method is shown in **Figure** **2**d,e. To obtain a cluster of self‐assembled Au NPs in the nanohole arrays, a small droplet of dense solution of colloidal Au Nanospheres (5.6 × 10^9^ particles mL^−1^) was injected between the moving substrate on the vacuum chuck and a stationary glass cover slide positioned about 500 µm above the substrate. As the substrate moves horizontally relative to a stationary glass slide, capillary forces at the liquid meniscus guide the Au NPs into the well‐defined nanohole of the membrane.^[^ ^18^ ^]^ During the particle placement experiments, the three parameters of substrate velocity, temperature, and glass slide height with respect to the substrate were controlled and continuously monitored. The initial selection of these parameters was based on pioneering studies in the CAPA domain.^[^ ^19^, ^20^ ^]^ The arrays were filled with monodisperse Au NPs (diameter = 150 nm). The Transmission Electron Microscopy (TEM) image of a single Au NP (Figure 2a), and Dynamic Light Scattering (DLS) measurements of Au NPs dispersed in water (Figure 2b), both confirm the 150 nm size of Au NPs. The Scanning Electron Microscopy (SEM) images of the formed assemblies, presented in Figure 2f(i,ii), demonstrate a high assembly yield, estimated to be over 99% across the holes of the interested area. In Figure 2f(ii), the typical morphology of the Au NPs accumulation on top of the membrane is shown, which consists of ≈10 layers. While the number of Au NP layers may vary slightly from batch to batch, the observed dynamics remained consistent, indicating that minor variations (e.g., 7 vs 12 layers) do not significantly impact molecular translocation behaviour. Furthermore, the tight attachment of Au NP layers significantly enhances the substrate stability and ensures consistent SERS performance even at high‐power laser measurements. To confirm this, we tested the stability of the device by repeatedly using it for multiple translocation experiments. After each experiment, the device was cleaned with oxygen plasma and subsequently monitored using SEM. The SEM images confirmed the resilience of the fabricated device, particularly in terms of the firm attachment of Au NPs within the nanohole arrays of the membrane. Additionally, to estimate the size of nanogaps, we have performed some calculations shown in Figure 2f(iii,iv) showcase the formation of miniaturized nanogaps with a transverse size below 10 nm. The details of the nanogap size calculation are provided in the Supporting Information (SI#1). We have also carried out Finite Element Method (FEM) simulations of the SERS enhancement factor in the gaps between Au NP dimers. The results, presented in Supporting Information (SI#1), demonstrate enhancement factors exceeding 10¹⁰ for a 7 nm gap (shown in Figure S1, Supporting Information). To further investigate the effect of interparticle distance, a parametric sweep was conducted over gap sizes ranging from 4 to 14 nm (shown in Figure S2, Supporting Information), confirming the expected exponential decay in enhancement with increasing separation. In other words, the SERS‐active region corresponds to the nanogaps within the holes, particularly those near the contact points between nanoparticles, as illustrated in Figure 2f(i). For gaps >7 nm, the plasmonic enhancement is insufficient to significantly contribute to the Raman signal.

The Ultraviolet‐Visible (UV‐Vis) spectrum of 150 nm Au NPs in aqueous solution, as shown in Figure 2c, exhibits a peak near 633 nm, making it well‐suited for Raman measurements using a laser with that wavelength (Figure 1). Since it is well known that individual 150 nm Au NPs support localised plasmon resonances at 532 nm, it is reasonable to assume that particle clustering causes the resonance to redshift. Remarkably, the system resonates in the whole visible range; however, when desired, the position of the resonance may be modulated by varying the nanoparticles' size. It should be mentioned that by the method proposed here, the densely packed cluster of Au NPs with the same size and morphology is feasibly deposited inside the nanohole arrays. This method demonstrates excellent performance in terms of simplicity, low cost, and robustness. However, improved control over the final gap size formed between the Au NPs inside the nano hole arrays is desirable, particularly in terms of size uniformity, as the current approach results in a relatively broad distribution of gap dimensions. In contrast, lithographic techniques allow for the fabrication of nanoscale gaps with much higher precision and reproducibility.^[^ ^21^, ^22^, ^23^ ^]^ For more detailed information, specialized reviews are available.^[^ ^24^, ^25^, ^26^, ^27^, ^28^ ^]^

### 2.3 Raman Measurements

Flow‐through single‐molecule Raman detection experiments were performed on a commercial microscope (InVia Renishaw) ad‐hoc modified with a SPAD camera (Laser 2000 SPC^3^ Series MPD SPAD 64 × 32 pixels, frame rate 96 kfps, median dark count rate of 100 cps, and a hold‐off time of 20 ns, photon detection efficiency 20% at 633 nm.), and a customized acquisition software.^[^ ^29^ ^]^ Moreover, the objective of the microscope was equipped with a custom electrophoretic system working across the developed plasmonic device. Under 100 mV bias applied across the nanopores, molecules are driven through the plasmonic nanopores. SERS measurements were performed under the 633 nm laser radiation in liquid phase (buffer solution NaCl 150 mm) with a 63x dipping objective. We estimate the overall efficiency of our experimental setup to ≈7%, meaning that we detect 1 photon over 14 really scattered by the molecule. However, to avoid confusion, we will refer to the number of photons collected without directly accounting for efficiency, except where necessary and explicitly stated.

**Figure 3:**
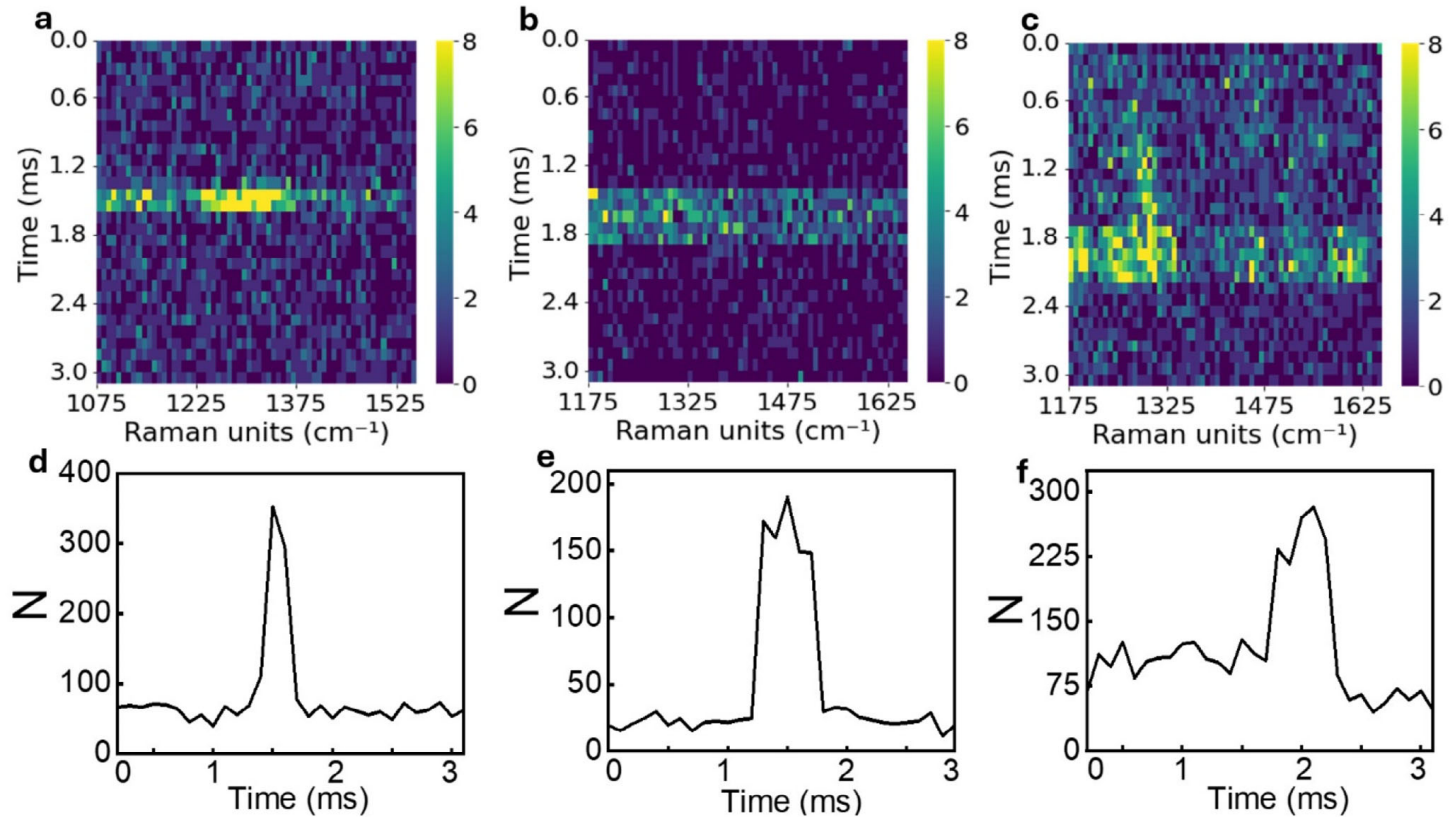
Representative Raman waterfalls illustrating translocated molecules of a) Poly‐Ala, b) Poly‐Arg, and c) Poly‐Lys at a concentration of 1 nm. Acquisition rate of 100 µs per frame. Plot of the total number of photons detected across the 64 × 32 pixels of the SPAD camera versus time, during a recording session lasting 3 ms, and involving translocation events of the three distinct molecules d) Poly‐Ala molecules, e) Poly‐Arg, and f) Poly‐Lys molecules.

We have translocated three distinct polypeptides: poly‐alanine (Poly‐Ala, length spanning from 10 to 50 AAs), poly‐arginine (Poly‐Arg, length spanning from 50 to 150 AAs), and poly‐lysine (Poly‐Lys, length spanning from 150 to 300 AAs). These polypeptides were selected for their diverse chemical structures and biological abundance. In the adopted buffer solution, these polypeptides are completely unfolded, thus exhibiting a linear shape. Since a single amino acid has a size of ≈0.35 nm, the chosen molecules have a length varying from a minimum of 3.5 nm (10 AA) up to 105 nm (300 AA). These dimensions allow the molecules to behave like flexible rods within the hotspot channel, adapting to the confined environment while maintaining their structural integrity. We successfully captured Raman spectra of these three polypeptides translocating into the pores with the acquisition time set to 100 µs. We chose this integration time in order to maximize the signal‐to‐noise ratio and discard very short events (<<100 µs) as explained thereafter. This way we can obtain robust statistics on the Raman scattering rate. Representative examples of the real‐time Raman waterfall spectra of the translocated polypeptides are presented in **Figure** **3**a–c, for a total recording time of 3 ms (30 consecutive acquisitions of 100 µs each with no interval in between). Figure 3d–f plots the total number of photons per frame (Raman signal intensity) versus the recording time for each molecule. The translocation of polypeptides can be clearly discriminated from the background. Regarding Figure 3d–f, the signal‐to‐noise ratio for Poly‐Ala, Poly‐Arg, and Poly‐Lys is ≈8, 9, and 4, respectively. The conventional Raman spectra of the polypeptides (supplier spectral database) and those acquired without the use of electrophoretic translocation are reported for completeness in Figure S3 (Supporting Information), where they are all compared. As expected, due to the different boundary conditions, the spectra are only partially comparable, reinforcing the importance of measurement conditions in interpreting Raman data. Further details on the Raman measurement protocols can be found in Supporting Note 3 (Supporting Information).

**Figure 4:**
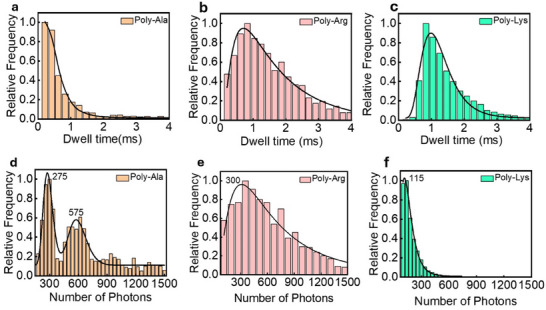
Normalized events’ rate histograms of three different polypeptides for a molecular concentration of 1 nm. Time frame of 100 µs. a–c) Dwell time distributions of Poly‐Ala, Poly‐Arg, and Poly‐Lys molecules, respectively. d–f) Statistics on the number of scattered photons for Poly‐Ala, Poly‐Arg, and Poly‐Lys molecules.

In addition to the Raman spectral analysis, we have also collected statistical data on the translocation events of the studied polypeptides, including metrics such as dwell time and photon scattering rate. This data is presented in **Figure** **4**. As expected, the shorter molecules tend to translocate faster (Figure 4a–c) while longer molecules take longer. By using the log‐normal distribution fitting model, we calculated the mean dwell‐time values, which are ≈300 µs, 700 µs, and 900 µs for Poly‐Ala, Poly‐Arg, and Poly‐Lys, respectively. The dwell time versus molecular length is presented in Figure S4 (Supporting Information) and does not exhibit a perfectly linear scaling with molecular length. This can be due to the different chemical and physical nature of the three molecules. Also, it is worth mentioning that short molecules are just a few nm in size. Being smaller or comparable to the size of the nanopores, they can pass with different orientations through the plasmonic spots. For example, a very short molecule of 5 nm can pass through the pore transversally. In those cases, the amino acids are not exposed to the plasmonic field in a sequential manner, and the translocation time is expected to be very short, namely in the order of a few microseconds.

To minimize the impact of this effect on our measurements, we set the acquisition time to 100 µs. This way we discarded undesired translocation dynamics that could lead to misinterpretation of data regarding photon scattering rate. This aspect confirms the importance of realizing nanopores with a geometry similar to biological pores, namely lateral size approaching 3 nm and pore length of 5–10 nm. As mentioned above, more established lithographic techniques may offer better control in this regard. According to our analysis, based on a few hundred events for each type of polypeptide, the average translocation time is 7 µs per amino acid. Notably, this value is significantly longer than the previously reported 0.46 µs per amino acid in solid‐state nanopores, making our process ≈15 times slower.^[^ ^12^ ^]^ In the referenced study, six different proteins were translocated through sub‐5 nm solid‐state nanopores under optimized conditions. The 15‐fold improvement in dwell time is likely attributed to the presence of Au NPs, which slow down molecular translocation by creating intricate pathways. This effect forces the molecules to navigate through a complex network, akin to the mechanism observed in gel electrophoresis.

We have analyzed the number of photons scattered from single molecules during the individual translocation events (Figure 4d–f). The mean photon scattering values were extracted by fitting the histogram in a log‐normal distribution fitting model. Interestingly, poly‐ala shows two distinct peaks at around 275 and 575 photons per translocation (Figure 4d). The origin of this double peak is not known; however, it could be due to a nonhomogeneous distribution of molecular weight in the sample that includes molecules with a length of 10 to 50 units. In contrast, Poly‐Arg and Poly‐Lys show a more conventional statistical distribution with a single peak at ∼300 and 115, respectively (Figure 4e,f). The recorded signals show an average of 6 photons per AA per translocation event. However, numbers are very different for individual AAs and fluctuate from one event to another since dwell time spans over an order of magnitude. According to the data, we figured out that the Raman scattering is: 14, 3, and 0.5 photons/AA for Poly‐Ala, Poly‐Arg, and Poly‐Lys, respectively. This is due in part to the fact that the homopeptides normalized to their unit of length, translocate with different speeds, with Poly‐Lys being the faster (4 µs AA^−1^) and Poly‐Ala the slower (10 µs AA^−1^). We will better analyse this behaviour thereafter. However, such a very different scattering rate suggests that for a real protein, the signal would be dominated by specific AAs whose Raman cross‐sections largely exceed those of the others. This finding is suitable for an approach in which the relative sequence of selected AAs is used to identify the proteins. In fact, according to bioinformatic studies^[^ ^30^, ^31^ ^]^ by measuring the relative sequence of only two types of AA, and neglecting all the other, >90% of the human proteins can be identified by comparing the recorded relative sequences with known sequences from the human proteome database.

Another important piece of information in Figure 4 is that the dwell time spans over an order of magnitude, consistent with extensive studies on both biological and solid‐state nanopores. This implies that the number of photons collected during individual translocations can vary significantly. To further investigate this aspect and to better quantify the scattering of the different molecules, we analysed the number of photons collected for each single event as a function of the corresponding dwell time. These data are presented in Figure S5 (Supporting Information). According to these graphs, there is a linear relationship between the dwell time and the number of photons scattered during the translocation. The slope of these graphs represents the photon scattering rate for each studied polypeptide. We found that Poly‐Ala shows a rate of 1286 photon ms^−1^, Poly‐Arg 398 photon ms^−1^, and Poly‐Lys 120 photon ms^−1^. Again, it is worth noting that the scattering rate of Poly‐Ala is more than 10 times higher than that of Poly‐Lys.

**Figure 5:**
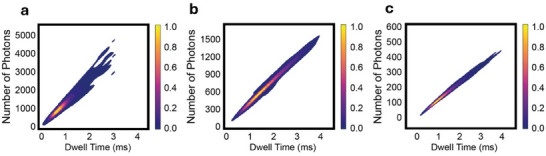
Kernel Density Estimation (KDE) heatmap showing the distribution of dwell time versus number of photons for translocation of a) Poly‐Ala, b) Poly‐Arg, and c) Poly‐lys molecules at a concentration of 1 nm and acquisition time of 100 µs. The color scale represents the normalized probability of events.

To complement the linear graphs depicting the relationship between dwell time and photon scattering, we have also generated Kernel Density Estimation (KDE) heatmaps illustrating the distribution of dwell time versus photon count for the polypeptide translocation. Data are reported in **Figure** **5**. Specifically, KDE heatmaps have been generated to provide a more detailed visualization of the distribution of dwell times and photon counts during the translocation of various polypeptides. These heatmaps offer a nuanced perspective, enhancing the understanding of the data beyond what is conveyed by the linear graphs alone.

As observed in Figure 5a, the distribution of data for Poly‐Ala molecules' translocation shows a relatively broad spread in both dwell time and photon count, indicating variability in translocation dynamics. Notably, the highest probability corresponds to an scattering of ≈200–500 photons and a dwell time of 0.3 ms. Accordingly, for Poly‐Ala molecules, a high photon scattering rate of 1286 photon ms^−1^ is found. For Poly–Arg molecule translocations (Figure 5b), the KDE map exhibits a more elongated and structured distribution, with a distinct peak around ∼0.7 ms and photon counts ranging from ∼250–750. These suggest that Poly‐Arg molecules generally translocate with a photon scattering rate of 395 photon ms^−1^. The KDE plot for Poly‐Lys molecules shows a narrower distribution, with a strong correlation between dwell time and photon count. The peak density showing the most probable events is around ∼0.9 ms with photon numbers up to ∼150, suggesting the lowest photon scattering rate of 120 photon ms^−1^.

Finally, we notice that the linear correlation between molecular length, dwell time, and photon scattering for all the peptides suggests that molecules progressively scatter Raman photons while passing through the hot spot. Such a constant scattering, in spite of unavoidable fluctuations, can be recorded over the noise background. Hence, these findings support the feasibility of sequential Raman readout of small molecular portions approaching the size of a few amino acids. Such a strategy may lead to protein identification by focusing the analyses on specific subsets of amino acids with higher Raman cross‐sections.^[^ ^30^, ^31^ ^]^

**Figure 6:**
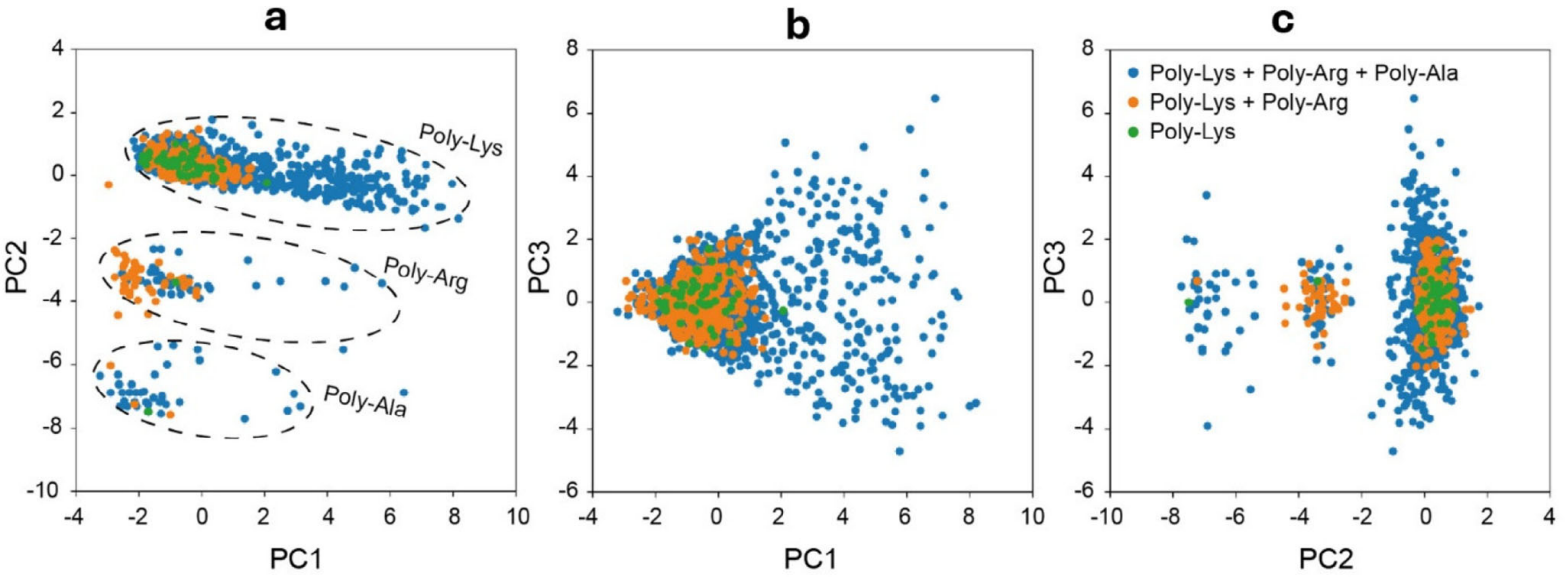
Principal Component Analysis (PCA) of sequential polypeptide addition. PCA plots illustrate the progression of system states upon sequential addition of poly‐amino acids. a) Separation of individual peptide contributions in PC1–PC2 space: Poly‐Lys (top), Poly‐Arg (middle), and Poly‐Ala (bottom) form distinct clusters. b) PC1–PC3 view shows overlapping distributions. c) PC2–PC3 projection highlights the temporal sequence: green (Poly‐Lys), orange (Poly‐Lys + Poly‐Arg), and blue (Poly‐Lys + Poly‐Arg + Poly‐Ala). Each new polypeptide addition leads to the emergence of a distinct cluster, reflecting its impact on system composition.

As a final discussion, polypeptide detection and discrimination at the single‐molecule level are further confirmed through an additional measurement in which the three analyte molecules were progressively introduced into the device. We first introduced and translocated Poly‐Lys, followed by the addition of Poly‐Arg, and finally Poly‐Ala. By the end of the experiment, all three molecules were present in the sample simultaneously. The data were examined by Principal Component Analysis, and the outputs are reported in **Figure** **6**. The three cases are represented by different colors in the PCA plot: green for Poly‐Lys, orange for Poly‐Lys + Poly‐Arg, and blue for Poly‐Lys + Poly‐Arg + Poly‐Ala. Each addition of a new molecule results in the appearance of a well‐defined cluster in PCA space. For example, in the PC3 versus PC2 projection (panel C), Poly‐Lys alone initially produces a green cluster on the right. Upon the addition of Poly‐Arg, a distinct cluster appears in the center. Finally, Poly‐Ala produces a cluster on the left. As expected, the number of points in the Poly‐Lys cluster is the largest since it was present for the longest duration. The Poly‐Arg cluster is smaller, and the Poly‐Ala cluster is the smallest. The overlap of the clusters is <1% hence we can preliminarily estimate the discrimination accuracy to be >99%. Overall, this experiment clearly demonstrates:

• The ability to discriminate individual amino acids even when delivered in a mixture.
• The single‐molecule nature of measurements. In fact, if two molecules were translocated simultaneously, their combined spectra would be indistinguishable and result in data points located between clusters. In such a scenario, the clusters would overlap or be connected by significant noise, which we do not observe.

## 3 Conclusion

This study presents a novel method for fabricating plasmonic nanopores via Capillary‐Assisted Particle Assembly and lithographic methods, producing cost‐effective, stable gold nanopores. The device provides narrow pathways containing multiple plasmonic hot spots for molecules translocating into the nanopores. The latter shows broad resonances covering most of the visible range with a tail in the NIR, thus being ideal for SERS experiments. Using electrophoresis, polypeptides of alanine, arginine, and lysine were translocated through the pores, and their Raman signals were recorded with a SPAD camera integrated into a Raman spectrometer. Notably, the method showed the ability to work with highly diluted samples corresponding to 1 nm. Results show a linear correlation between translocation time, photon scattering, and molecular length, with an average translocation time of ≈7 µs per amino acid and an average photon scattering of ≈6 photons per amino acid. Specifically, Poly‐Alanine molecules can scatter a high number of photons (with the photon scattering rate of 1286 ms^−1^) that dominate over the others. The efficiency of our setup is currently 7%, meaning the actual number of scattered photons is 14 times higher. This presents a strong opportunity for enhancing signal intensity. When combined with optimized protocols for nanopore fabrication and molecular translocation, this approach could pave the way for single‐molecule identification through Raman sequential readout. Given that the scattering of certain amino acids dominates over others, the most reliable strategy appears to be focusing on the relative sequence of those amino acids that produce the strongest signals.

## 4 Experimental Section

### Chemicals and Materials

Three different kinds of polypeptides: poly‐alanine (Poly‐Ala, length spanning from 10 to 50 AAs), poly‐arginine (Poly‐Arg, length spanning from 50 to 150 AAs), and poly‐lysine (Poly‐Lys, length spanning from 150 to 300 AAs) were purchased from Sigma‐Aldrich. Gold nanoparticles with a diameter of 150 nm (5.6 × 10^9^ particles mL^−1^) were purchased from Sigma‐Aldrich.

### Solid‐State Nanopore Fabrication

In this work, the free‐standing Si_3_N_4_ membranes with 100 nm height, 50 mm width, and 50 mm length were manufactured by processing the Si [100] wafer coated by 100 nm Si_3_N_4_ on both sides using low‐pressure chemical vapor deposition (LPCVD). The processing involves a standard protocol of UV lithography, reactive ion etching, and KOH wet etching. Afterward, an oxygen plasma treatment of the surface was applied (plasma machine: Electro‐Technic Products Inc., 100 W, 3 min). Then, by sputtering of 10 nm Ti as an adhesion layer and subsequently a 20 nm Au layer, focused ion beam (FIB) drilling was employed to drill nanopores of 190 nm in diameter on the suspended membranes. The milling process was carried out using a Helios Nanolab 650 FEI dual‐beam system. The 5 × 5 square patterns of holes were milled with the parameters of 30 kV voltage, milling current of 18 pA, and a dwell time of 80 ms. This current was selected after calibrating the beam energy to ensure the formation of circular pores without distorting the single SiN membrane layer.

### Raman Spectroscopy

SERS spectra were collected on a Renishaw inVia commercial confocal Raman microscope, equipped with a CW DPSS laser with a wavelength of 633 nm.

The SPAD camera is based on a 32 × 64 single photon detector array designed by Politecnico di Milano that can reach a 96 kfps frame rate.

### Statistical Analysis

A find_peaks function in Python is employed to identify peaks corresponding to translocation events. By analyzing the number of events and their associated dwell times, an exponential distribution was observed, suggesting a range of events weighted by their occurrence (from short to long translocation durations). A logarithmic fit of the relative frequency versus dwell time yielded a distinct peak for each group of events, representing the most probable dwell time. Assuming an average molecular length, the average dwell time was divided by this length to estimate the average dwell time per amino acid (AA).

To assess the validity of the data analysis method for statistical data interpretation, the Pearson correlation coefficient (r) was calculated for the two data sets: dwell time and number of photons for each polypeptide. For all three groups of data related to the translocation event of Poly‐Ala, Poly‐Arg, and Poly‐Lys molecules, the obtained r is close to 1, demonstrating a high correlation between dwell time (T) and the number of photons (I).

## Conflict of Interest

The authors declare no conflict of interest.

## Supporting information



Supporting Information

## Data Availability

We will post the manuscript in a public repository in the coming weeks. If the manuscript is accepted for publication in Advanced Materials, we will opt for the open‐access model if financially sustainable.
