# Supplementary material for: High‐Speed Raman Readout of Single Polypeptides via Plasmonic Nanopores
Source: Adv Mater. 2025 Jul 9;37(39):2504436. doi: 10.1002/adma.202504436 (PMC12506624; doi:10.1002/adma.202504436)
Supplement: Supplementary file 1 — Supporting Information [file ADMA-37-2504436-s001.docx]

**Supporting Information**

**High-Speed Raman Readout of Single Polypeptides Via Plasmonic Nanopores**

Foroogh Khozeymeh Sarbishe^1^, Kirill Khabarov^1^, Maria Blanco Formoso^1^, Ilaria Micol Baldi^1,4^, Veronica Storari^2^, Henri Haka^2^, Massimo Mastrangeli^3^, Francesco Difato^1^, Andrea Armirotti^1^, Federica Villa^2^, Francesco Tantussi^1^, and Francesco De Angelis^1^

^1^ Istituto Italiano di Tecnologia, Via Morego 30, 16163, Genova, Italy

^2^ Department of Electronics, Information and Bioengineering, Politecnico di Milano, Via G. Ponzio 34/5, Milano 20133, Italy

^3^ Department of Microelectronics, Delft University of Technology, 2628CD Delft, the Netherlands

^4^ Department of Physics, University of Genoa, Via Dodecaneso 33, 16146 Genoa, Italy.

* Corresponding author: [francesco.deangelis@iit.it](mailto:francesco.deangelis@iit.it)

**Table of contents:**

**Supporting Note 1** | Nanopore size calculation.

**Supporting Note 2** | SERS Enhancement Factor simulations.

**Supporting Figure S1**| SERS electromagnetic enhancement factor at a wavelength of 633 nm in an aqueous environment for a gold dimer cluster composed of nanoparticles with a radius of 150 nm. (a) Enhancement factor at a fixed interparticle gap of 7 nm and (b) Enhancement factor as a function of gap distance ranging from 4 nm to 14 nm, presented on a logarithmic scale.

**Supporting Figure S2**| Electromagnetic field enhancement (|E|⁴) in nanoparticle dimers as a function of gap size. Simulated |E|⁴ enhancement maps are shown for dimer gaps of (a) 4 nm, (b) 6 nm, (c) 8 nm, (d) 10 nm, (e) 12 nm, and (f) 14 nm. As the gap increases, the field enhancement decreases significantly, indicating a strong dependence of plasmonic coupling on interparticle spacing. The colour scale represents the magnitude of the local |E|⁴ enhancement, highlighting the hotspot region between the particles.

**Supporting Note 3** | Raman measurements.

**Supporting Figure S3** | Raman and SERS reference spectra for the target molecules: Poly-Ala, Poly-Arg, and Poly-Lys. (a–c) Standard Raman reference spectra for Poly-Ala, Poly-Arg, and Poly-Lys, respectively, obtained from the supplier (Sigma-Aldrich). (d–f) Experimentally acquired SERS spectra for 1 mM solutions of the same molecules, measured on a gold substrate coated with 150 nm gold nanoparticles using a 633 nm excitation laser (Renishaw system, 2–4 min integration time). The spectra were averaged and normalized between 0 and 1. (g–i) SPAD-based SERS spectra for Poly-Ala, Poly-Arg, and Poly-Lys, measured at 1 nM concentrations with 100 µs acquisition time. Amide I and Amide II bands are highlighted, showing consistency with the reference spectra and enabling identification of the target molecules.

**Supporting Note 4 |** Statistics on Data.

**Supporting Figure S4 |** Linearity found between dwell time and (a) molecular length and (b) molecular weight, (c) Dwell time versus the molecular weight of the molecules investigated in Ref. [12], and (d) comparison of our results with results of Ref. [12].

**Supporting Figure S5** | Correlation between dwell time and number of photons. (a-c) graph of number of scattered photons in terms of Dwell time of Poly-Ala, Poly-Arg, and Poly-Lys molecules translocation, the solid line is a linear fit

**Supporting Note 5 |** Data Analysis.

**Supporting Table 1.** Summary of analyte molecules, their length ranges (in amino acids), corresponding ranges of calculated dwell time per amino acid (µs/AA), and corresponding photon scattering ranges.

**Supporting Table 2.** Calculated Pearson correlation coefficients for the target polypeptides, indicating the strength of the linear relationship between dwell time and number of photons during the translocation events.

**Supporting Note 1. Nanopore size calculation**.

To determine the nanopore size, the Fast Fourier Transform (FFT) process was applied to the SEM image of the nanopores. Using the FFT bandpass filter, horizontal stripes were suppressed. By applying an appropriate threshold, only transverse areas were considered for analysis.

**Supporting Note 2. SERS Enhancement factor simulations.**

We have performed 3D finite element method (FEM) simulations using COMSOL Multiphysics to evaluate the electromagnetic field distribution and SERS enhancement factors within the nanogap formed by a dimer of gold nanoparticles (radius 150 nm) in an aqueous environment. These simulations were conducted at a wavelength of 633 nm, which matches the experimental excitation condition. Our results demonstrate a strong localization of the electric field (hot spot) in the gap region, with calculated electromagnetic enhancement factors (|E|⁴) exceeding 10¹⁰ for a 7 nm gap. Clearly, these values refer to ideal conditions while in the practical case they are expected to be lower. We further explored how this enhancement varies with the interparticle distance, performing a parametric sweep over gap sizes from 4 nm to 14 nm, confirming the expected exponential decay in enhancement with increasing separation. The corresponding field distributions and EF plots have now been shown in Figures S1 and S2.


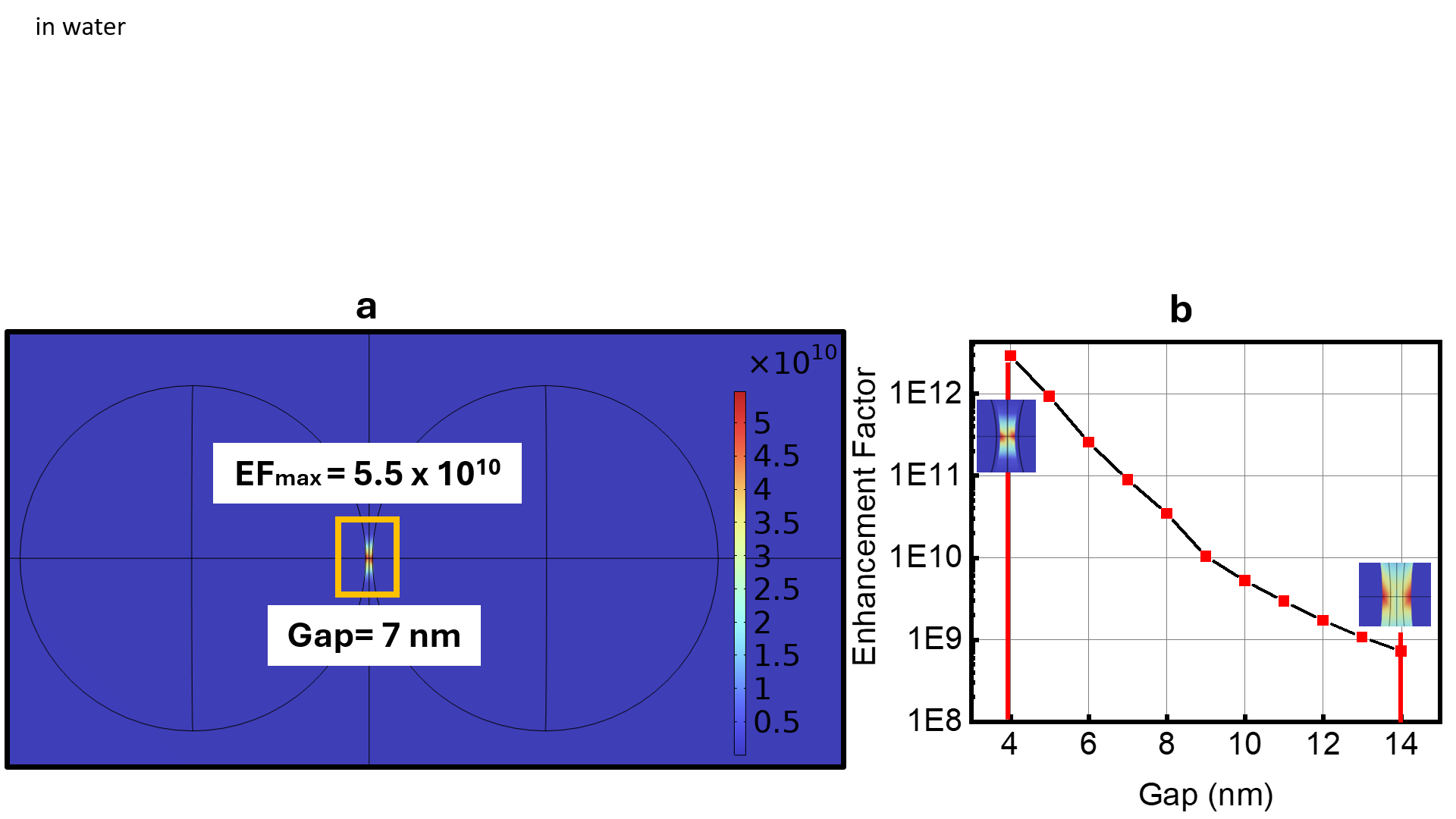
 ***Supporting Figure S1.*** *SERS electromagnetic enhancement factor at a wavelength of 633 nm in an aqueous environment for a gold dimer cluster composed of nanoparticles with a radius of 150 nm. (a) Enhancement factor at a fixed interparticle gap of 7 nm and (b) Enhancement factor as a function of gap distance ranging from 4 nm to 14 nm, presented on a logarithmic scale.*


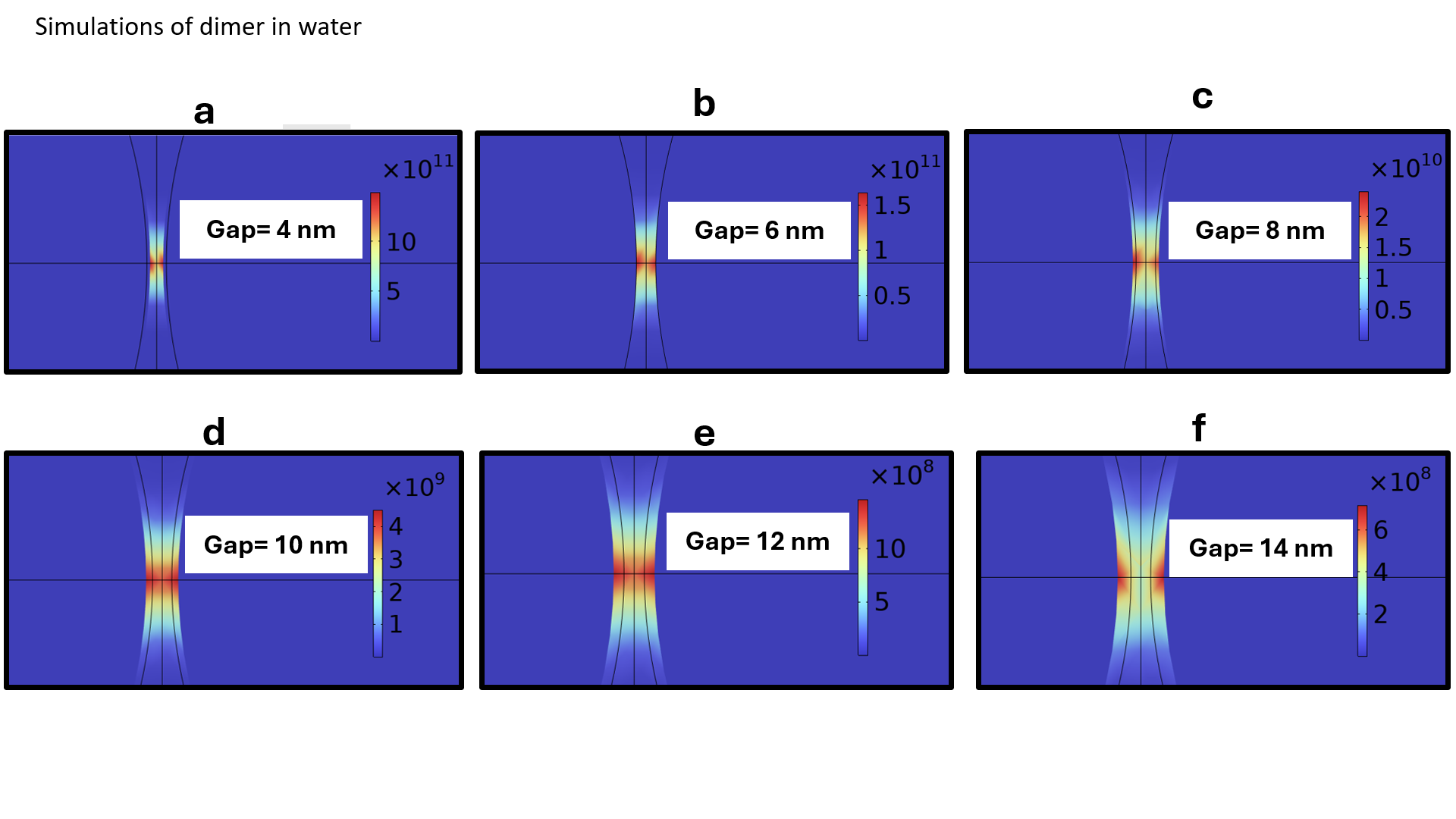


***Supporting Figure S2.*** *Electromagnetic field enhancement (|E|⁴) in nanoparticle dimers as a function of gap size. Simulated |E|⁴ enhancement maps are shown for dimer gaps of (a) 4 nm, (b) 6 nm, (c) 8 nm, (d) 10 nm, (e) 12 nm, and (f) 14 nm. As the gap increases, the field enhancement decreases significantly, indicating a strong dependence of plasmonic coupling on interparticle spacing. The colour scale represents the magnitude of the local |E|⁴ enhancement, highlighting the hotspot region between the particles.*

**Supporting Note 3. Raman measurements**

The corresponding SERS spectra have been recorded and are presented in Supporting Figure S3 (g-i). For validation, these spectra are compared with both the standard Raman reference spectra provided by the supplier (Sigma-Aldrich; Figure S3(a–c)) and experimentally obtained SERS reference spectra (Figure S3(d–f)). To obtain SERS reference spectra we performed Raman measurements using 1 mM solutions of Poly-Ala, Poly-Arg, and Poly-Lys. For each sample, a droplet of the respective solution was deposited onto a gold substrate coated with multiple layers of 150 nm gold nanoparticles. Raman spectra were collected using a 633 nm excitation laser on our Renishaw system, with acquisition times ranging from 2 to 4 minutes and a CMOS camera for detection. The spectra obtained in the 1200–1600 cm⁻¹ range were summed, averaged, and normalized (scaled between 0 and 1) to produce representative SERS reference spectra, which are shown in Supporting Figure S3 (d–f).

The integration of supplier-provided reference spectra with newly acquired experimental SERS spectra, and the SPAD-based results offers a comprehensive and reliable identification of the characteristic spectral features of the target molecules.


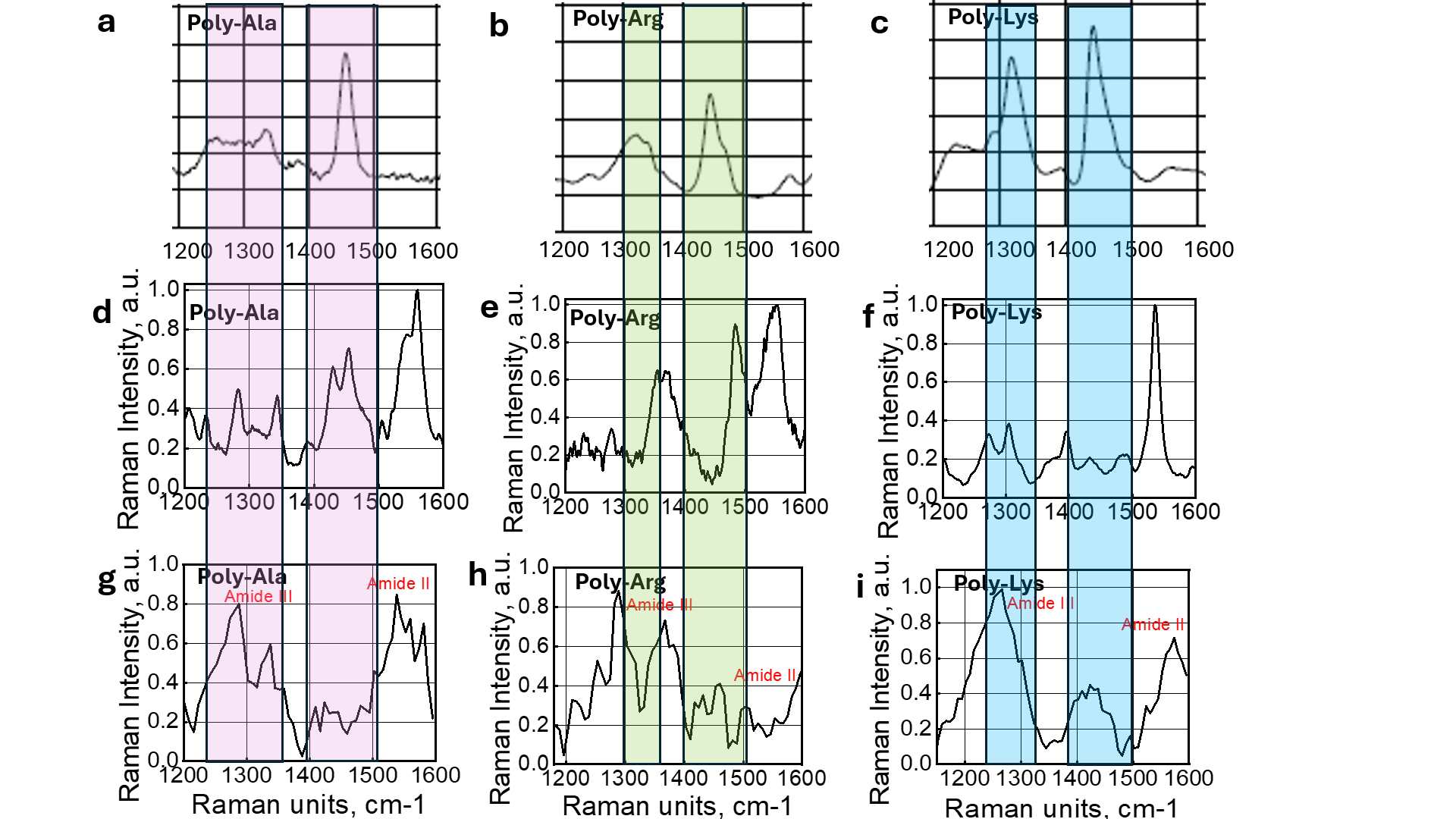


***Supporting Figure S3****. Raman and SERS reference spectra for the target molecules: Poly-Ala, Poly-Arg, and Poly-Lys. (a–c) Standard Raman reference spectra for Poly-Ala, Poly-Arg, and Poly-Lys, respectively, obtained from the supplier (Sigma-Aldrich). (d–f) Experimentally acquired SERS spectra for 1 mM solutions of the same molecules, measured on a gold substrate coated with 150 nm gold nanoparticles using a 633 nm excitation laser (Renishaw system, 2–4 min integration time). The spectra were averaged and normalized between 0 and 1. (g–i) SPAD-based SERS spectra for Poly-Ala, Poly-Arg, and Poly-Lys, measured at 1 nM concentrations with 100 µs acquisition time. Amide I and Amide II bands are highlighted, showing consistency with the reference spectra and enabling identification of the target molecules*.

For Poly-Ala molecules, the Raman spectrum in panel (a) shows a double-peak feature centered around ~1300 cm⁻¹, as well as another distinct peak near ~1450 cm⁻¹. These peaks are also present in the SERS reference spectrum (panel d), though with slight broadening and some variation in relative intensity. The SPAD-based spectrum (panel g) clearly exhibits peaks in the same regions, particularly around ~1300 cm⁻¹, corresponding to the Amide III band. This indicates that the SPAD-based SERS measurements successfully capture the key vibrational modes of Poly-Ala. Notably, a peak around ~1550 cm⁻¹, likely corresponding to the Amide II band, appears in both SERS spectra (panels d and g), but is either absent or extremely weak in the Raman reference spectrum (panel a), suggesting possible enhancement or activation under SERS conditions.

For Poly-Arg, the Raman spectrum (panel b) features a broad peak slightly above ~1300 cm⁻¹ and a dominant peak between 1400 and 1500 cm⁻¹. These features are also observed in the SERS reference spectrum (panel e), with a minor red shift. The SPAD-based spectrum (panel h) retains both features with reasonable agreement in peak position and relative intensity. This confirms that SPAD-based SERS preserves the characteristic spectral fingerprints of Poly-Arg aligns well with both the standard Raman and SERS reference spectra. For Poly-Lys molecules (panels c, f, i), the Raman spectrum (panel c) displays sharp peaks near 1300 cm⁻¹ and 1450 cm⁻¹. The SERS reference spectrum (panel f) shows these same peaks with slight spectral shifts. In the SPAD-based spectrum (panel i), these two features are again clearly resolved, further supporting reliable detection of Poly-Lys. Good agreement across all three spectral methods demonstrates high confidence in the molecular identification. These observations underline the high sensitivity of this Raman technique, demonstrating its ability to produce clear and distinct spectra at the single-nucleobase level, even when using very low molecular concentrations. This highlights the potential of the method for further development and refinement in the analysis of polypeptides. However, it is worth mentioning that in the flow-through configuration, small changes in peak intensities or positions may indicate structural alterations. Stress from the flow-through configuration may also induce shear forces, altering backbone vibrations in the Amide III and II regions or leading to partial unfolding of the polypeptides. Additional factors, including van der Waals forces and hydrophobic interactions with nanoparticle surfaces, could influence the ability of molecules to pass through the system, either facilitating or hindering movement. Variations in Raman hotspots may further affect the precise peak positions of chemical groups in the polypeptides.

**Supporting Note 4. Statistics on Data**

The dwell time, which represents the duration each polypeptide spends within the detection region during translocation, provides critical insights into the dynamics of molecular interactions and transport behavior. Here as shown in Figure 4 (a-c) of the manuscript, the dwell time distribution reveals the most probable durations of 200-400 µs, 600-800 µs and 800-1000 µs for the respectively Poly-Ala (10-50 AAs), Poly-Arg (50-150 AAs), and Poly-Lys (150-300 AAs) molecules, respectively. As an approximation we consider the average length of 30 AA for Poly-Ala, 100 AA for Poly-Arg and 225 AA for Poly-Lys. Based on our analysis, we observe a linear correlation between the dwell time of analyte molecules and both their length (in amino acid units) and molecular weight (in kDa). This relationship is depicted in Figure S4(a-b), where the dwell time exhibits a proportional increase with molecular length, suggesting that longer polypeptides experience extended translocation durations. Similarly, the linear dependence of dwell time on molecular weight implies that larger proteins require longer translocation times. Furthermore, Figure S4(c) presents the dwell time as a function of molecular weight for three different proteins examined in Ref. [12]. Compared to their findings, our data demonstrates a significant enhancement in dwell time, as illustrated in Figure S4(d). This notable increase highlights the influence of our nanopore design on the translocation dynamics, further supporting a size- and weight-dependent translocation mechanism.


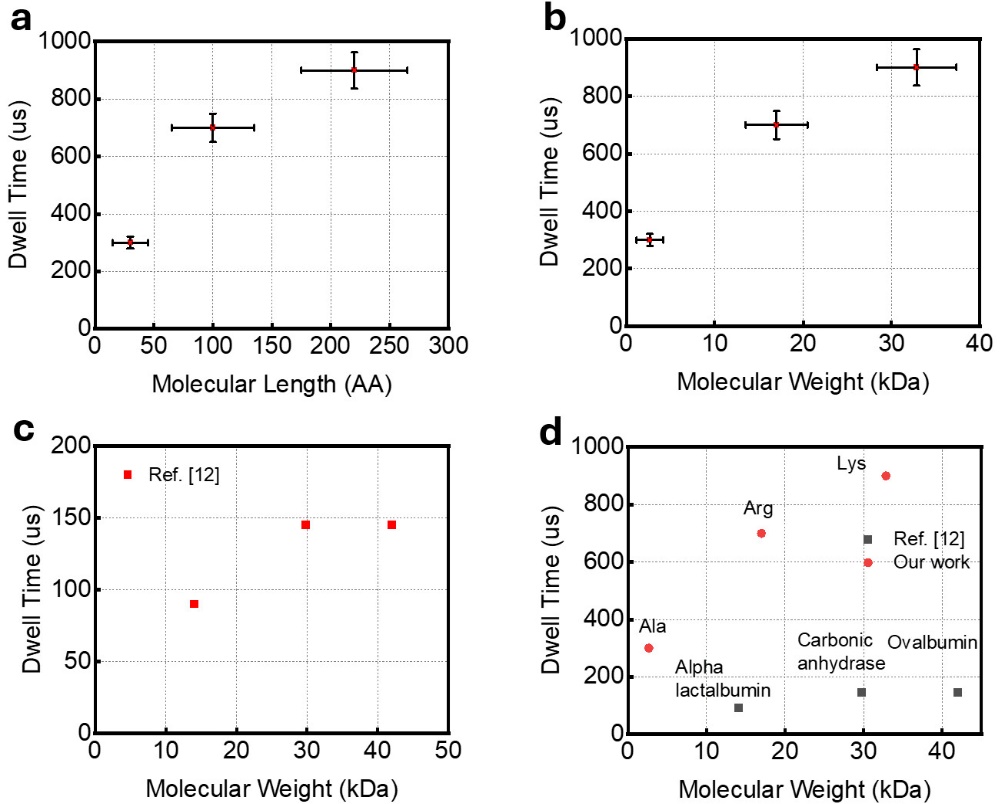


***Supporting Figure S4.*** *Linearity found between dwell time and (a) molecular length and (b) molecular weight, (c)Dwell time versus the molecular weight of the molecules investigated in Ref. [12], and (d) comparison of our results with results of Ref. [12].*

Furthermore, the photon distribution analysis captures the variability in Raman signal intensity during individual translocation events. We figured out that the Raman scattering of poly-alanine is the strongest while poly-lysine is weakest. By correlating photon distributions with dwell times, we can infer relationships between molecular conformation and the efficiency of translocation under the experimental setup. To achieve this, we have extracted the corresponding pair of dwell time and number of photons for each translocation event. This data is presented in Figure S5 (a-c).


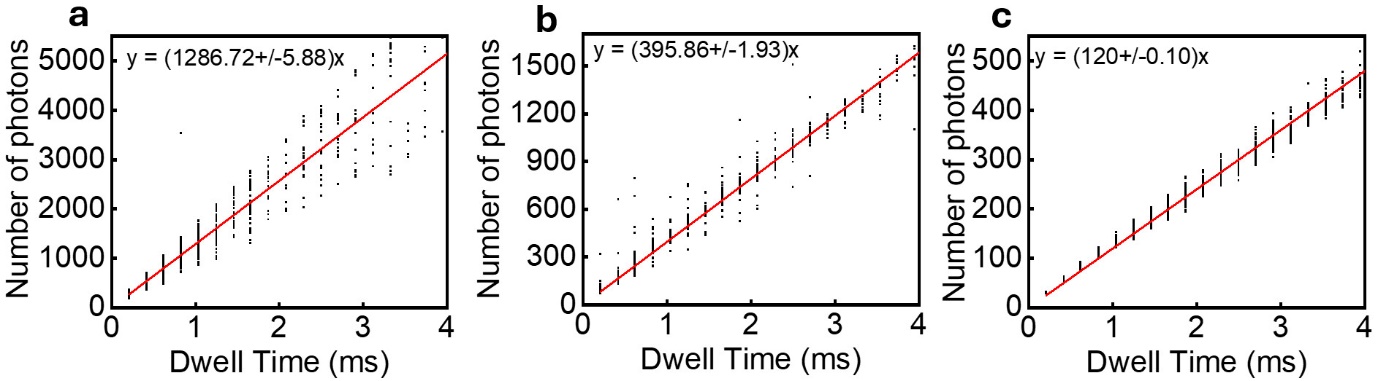


***Supporting Figure S5.*** *Correlation between dwell time and number of photons. (a-c) graph of number of scattered photons in terms of Dwell time of Poly-Ala, Poly-Arg, and Poly-Lys molecules translocation, the solid line is a linear fit.*

As can be seen in Supporting Figure S5, there is a linear relationship between the dwell time and number of photons scattered during the translocation. The slope of these graphs represents the photon scattering rate for each studied analyte. This parameter is highest for Poly-Ala (1286/ms) and lowest for Poly-Lys (120/ms), with an intermediate value of 395/ms for Poly-Arg molecules.

These statistical metrices offer a comprehensive perspective on the behavior of polypeptides during translocation, complementing the detailed vibrational information obtained from Raman spectra. Furthermore, the statistics on measured data does confirm the extreme sensitivity of the SERS substrates that can produce clear and distinguishable spectra. These measurements significantly enhance our understanding of the structural dynamics and interactions of Poly-Ala, Poly-Arg, and Poly-Lys under flow-through conditions.

**Supporting Note 5. Data Analysis**

As stated in the main text we found an average transaction time of 7 us per amino acid. This value was achieved as follows. We considered all translocation events (i.e., all molecules) and normalized the measured dwell time by the average number of amino acids in each molecule. Specifically, we divided the dwell time of Poly-Ala by 30, that of Poly-Arg by 100, and that of Poly-Lys by 225. We then averaged all these normalized values. The resulting estimate, approximately 7 µs, represents the average residence time of a single amino acid within the plasmonic hotspot.

It is important to note that significant variability exists from one event to another, as shown in Figure 4(a–c) of the main text, which presents the dwell time distributions for each molecule. This variability is expected. In both biological and solid-state nanopore literature, it is well established that the dwell time of individual DNA strands can span one to two orders of magnitude.

More in details the data were analyzed as follows. A find peaks function in Python is employed to identify peaks corresponding to translocation events. By analyzing the number of events and their associated dwell times, we observed an exponential distribution, suggesting a range of events weighted by their occurrence (from short to long translocation durations). A logarithmic fit of the relative frequency versus dwell time yielded a distinct peak for each group of events, representing the most probable dwell time. Assuming an average molecular length, we divided the average dwell time by this length to estimate the average dwell time per amino acid (AA). However, this value should also be normalized by the minimum and maximum lengths of the molecules to determine the full range of dwell times. The updated calculations for each molecule are presented in Table 1. In addition, the corresponding photon scattering ranges have also been included in Table 1.

**Table 1.** Summary of analyte molecules, their length ranges (in amino acids), corresponding ranges of calculated dwell time per amino acid (µs/AA), and corresponding photon scattering ranges.

| Analyte molecule | Molecule Length (AA) | Dwell Time Range (µs/AA) | Number of Photons Range |
| --- | --- | --- | --- |
| Poly-Ala | 10-50 | 30-6 | 42.5-8.5 |
| Poly-Arg | 50-150 | 14-4.6 | 6-2 |
| Poly-Lys | 150-300 | 6-3 | 0.76-0.38 |

Finally, it is important to note that every method of analysis involves approximations. To assess the validity of our data analysis method for statistical data interpretation, we calculate the Pearson correlation coefficient (r) [1, 2] for the two data sets: dwell time and number of photons for each polypeptide. The formula for the Pearson correlation coefficient (r) is expressed as follows:

$$r= \frac{\sum_{i=1}^{n} \left( T_{i}-\bar{T} \right) \left( I_{i}-\bar{I} \right)}{\sqrt{\sum_{i=1}^{n} \left( T_{i}-\bar{T} \right)^{2}\sum_{i=1}^{n} \left( I_{i}-\bar{I} \right)^{2}}}$$

Here T_i_ represents the *i-*th value of dwell time variable, I_i_ is the i-*th* value of Intensity (number of photons), $\bar{T}$ is the mean of variable T, and $\bar{I}$ is the mean of variable I. The parameter n indicates the number of data points. The results of r calculation have been presented in Table 1. For all the three sets of data related to translocation event of Poly-Ala, Poly-Arg and Poly-Lys molecules, the obtained r is close to 1 demonstrating a hight correlation between dwell time (T) and the number of photons (I).

**Table 2.** Calculated Pearson correlation coefficients for the target polypeptides, indicating the strength of the linear relationship between dwell time and number of photons during the translocation events.

| Analyte molecule | Molecule Length (AA) | Pearson Coefficient |
| --- | --- | --- |
| Poly-Ala | 10-50 | 0.98 |
| Poly-Arg | 50-150 | 0.98 |
| Poly-Lys | 150-300 | 0.99 |

**References**

[1] E. A. Pankrushina, A. S. Kobuzov, Y. V. Shchapova, S. L. Votyakov, *J. Raman Spectrosc.* **2020**, *51*, 1549–1562.

[2] B. Kozma, A. Salgó, S. Gergely, *J. Chemom.* **2023**, *37*, e7005.
